# A Psychological Perception Mechanism and Factor Analysis in Landsenses Ecology: A Case Study of Low-Carbon Harmonious Discourse

**DOI:** 10.3390/ijerph18136914

**Published:** 2021-06-28

**Authors:** Lan Zhang, Guowen Huang, Yongtao Li, Shitai Bao

**Affiliations:** 1 School of Foreign Studies, South China Agricultural University, Guangzhou 510640, China; 2 The College of Natural Resources and Environment, South China Agricultural University, Guangzhou 510640, China; yongtao@scau.edu.cn (Y.L.); bst100@scau.edu.cn (S.B.)

**Keywords:** landsenses ecology, harmonious discourse analysis, ecolinguistics, linguistic landsense, ecological self

## Abstract

Landsenses ecology has been widely applied in research into sustainable consciousness and behavior and the notion of landsense creation realizes the unity of the macro physical senses and micro psychological perceptions. However, a great deal of current research about landsenses ecology has concentrated on the dimension of the physical senses, while there have been relatively few studies on the dimension of its psychological perception. This paper begins by clarifying the concept of self and explaining out that the psychological perception mechanism of landsense creation represents a process of guiding people to know themselves and realize their ecological self. It then utilizes the example of low-carbon discourse to explore the factors contributing to the resonance of ecological self-vision. Our results show that the perceived self-efficacy, environmental concern and environmental knowledge triggered by ecological discourse are the main factors contributing to the resonance of sustainable vision, thus clarifying the indicators of landsenses ecology at the level of psychological perception. Our purpose is to effectively guide the landsense creation of harmonious discourse and promote people to engage in potentially more sustainable behavior.

## 1. Introduction

Landsenses ecology is a scientific system that integrates landscape ecology with people’s vision and social needs. Its ideas mainly come from the practice of land-use planning, eco-environmental development, restoration of damaged ecological environments, improvement of ecological services, etc., and at the same time it serves to guide and inform these practices [1]. Landsenses are mainly represented by two kinds of indexes: physical senses and psychological perceptions. At present, while there has been some research published on physical sense indexes, research into the psychological perceptions of landsenses ecology is relatively weak and a comprehensive index system has not yet been established [2]. From the perspective of harmonious discourse analysis [3,4], this paper analyzes the micro-process of constructing low-carbon consciousness in low-carbon publicity discourse, which can make up for the gap in the psychological perception index of landsenses ecology. It then carries out an exploratory factor analysis on the elements of psychological perception caused by ecological discourse in order to explore the key influencing factors of ecological consciousness. This can provide informational support for the landsense creation of ecological harmonious discourse, so as to effectively achieve vision resonance in ecological harmony at the level of cognition, and to promote the social behavioral norms of ecological harmony at the level of practice.

## 2. Literature Review and Research Hypothesis

### 2.1. The Evolution and Connotations of the Concept of Self

#### 2.1.1. Id, Ego and Superego

Freud divided people’s mental activities into different levels of consciousness, including conscious, pre-conscious and unconscious (also known as subconscious), regarding the sum of mental activities as a dynamic energy system, namely the personality. Freud divided personality into id, ego and superego [5]. In Freud’s view, the id is the most primitive part of the personality and represents basic needs and impulses, forcing people towards certain immoral or forbidden dreams and desires. The superego is the moral part of the personality that is formed in the process of accepting the moral norms of the social culture. It plays a controlling role in the personality structure to ensure that some unwanted desires do not appear or, if they do appear, only appear in an acceptable form. The ego is differentiated and developed from the id and represents a kind of coordination and accommodation when the various needs of the id cannot be met immediately in reality. The id embodies the biological instinct of human beings that acts according to the Pleasure Principle and is “the primitive person”. The superego is governed by the Perfection Principle, represents ideals and conscience and is “the moral person”. The ego coordinates the demands of the id and superego in a satisfactory and responsible manner according to the Reality Principle. “The realistic person” is the ego, which cushions and mediates between the impulses of the id and the controls of the superego. In the structure of the personality, id, ego and superego interweave with one another to perform their respective duties and thus form an organic whole.

#### 2.1.2. Self

The term “self” was first put forward in a psychological context by the American thinker William James (1890) [6]. From the viewpoint of biological instinctive determinism, James regarded the selfish impulse as the core of the self, and believed the self to be a kind of consciousness and psychological process, as well as one’s observation and cognition of one’s own existing state and characteristics. James divided the self into “me” as the experience object and “I” as the actor. As the experiential object, “me” (i.e., the Empirical Self) has a hierarchical structure and can be divided into three levels from low to high: material self, social self and spiritual self. The material self refers to a real object, a person or a place, including the sense of one’s own body and the perception of the things one owns. Social self refers to how we are perceived and recognized by others, including the various social positions we hold and the various social roles we play. Spiritual self refers to the internal psychological quality we perceive, which represents our subjective experience of ourselves, such as abilities, attitudes, emotions, interests, motives, opinions, traits, desires, self-confidence, conscience, guilt and so on, which are all components of the spiritual self.

Different forms of self would appear in different situations. Gergen, a representative of social constructivism, used “saturated self” to describe the diversity of the self and believed that the concept of self should be removed from the mind and placed in the domain of social discourse [7]. The self can be recognized and developed by interaction with the environment and has characteristics of both stability and development.

#### 2.1.3. Ecological Self

Naess (1985) [8] proposed the concept of ecological self and argued that self-maturity went through three stages: from ego to social self and then to metaphysical self. Naess used the concept of ecological self to represent the metaphysical self and thought that a person’s ecological self was an individual identification that could be thought of as our first formative stage in nature. Social and interpersonal relationships are important, but we are much richer in our physical relationships not only with other people and the human community but also with other living things [9]. The self in the Western tradition is a specific individual person, a lowercase *self*, while the ecological self is a self combined with ecological consciousness that is closely related to the surrounding environment and is distinguished as the capital *Self* [5]. The process of self-maturation is the process of our identification with others and the process of self-realization is the process of expanding and deepening the self. The deep ecology represented by Naess advocates self-realization. Continuous self-realization will mean the expansion and deepening of the self no matter how different individuals are [9].

#### 2.1.4. The Harmonious Connotation of the Concept of Self

A review of studies related to self reveals that the conception features both a diachronic evolution and a synchronic expansion. At different stages, theories of self have been recognized and followed but deficiencies have also been identified and supplemented. For example, when Freud was considered to be concerned with the self as the core and others criticized his inadequate consideration of the relationship between the self and society [10], research into the concept of self at the social level emerged. When the social constructivist view of the self was considered to suggest only that the structure of self should be expanded to include the social aspects of the global environment, but not to include nature in its scope [5], the concept of ecological self came into being.

In this paper, the authors hold that although theories of self at different stages have emphasized different perspectives, the core pursuit of all theories of self is harmony, whether from the individual, social or ecological level. Freud sought the harmony of his inner threefold self on the level of the individual so as to achieve harmony with external social norms. The various social constructionist theories of self are committed to the unity of activity and regulation, which is the pursuit of harmony between the essential self and relational self at the social level. The concept of ecological self claims that the self gradually expands beyond the human category and reaches an overall identity with the non-human world, i.e., the realm of the unity of man and nature. It can be observed that these concepts of self themselves exist in harmony and their varying kinds of significance also represent the pursuit of a form of harmony.

### 2.2. The Psychological Perception Mechanism of Landsense Creation

The concept of landsenses ecology holds that, “in practice, people usually endow or integrate one or more of their visions into a carrier through appropriate forms of manifestation, so that others (including themselves) can graft these visions from this carrier and associated forms of manifestation. These visions can guide or regulate people’s words and deeds, and further promote the realization of sustainable development” [11]. It further stresses that, “these carriers can be hardware, such as buildings, gardens, cities and blocks, as well as cultural resources such as poetry, novels, paintings, advertisements and songs” [12]. A carrier with these kinds of attributes is called a landsense and the whole process of conception and construction of the landsense is known as landsense creation. Research into the theory and methodology of landsense creation belongs to a new discipline called general landsenses ecology [11]. A landsense is represented by both physical senses and psychological perceptions [2]. Physical senses include vision, hearing, taste, smell, touch and other dimensions, while psychological perceptions include safety, culture, ethics, ecology and so on. The psychological perception mechanism involved in landsense creation can be summarized as the process of self-cognition. The self is not only an object of perception but also a subject with agency. The individuals’ behavior toward the environment varies with their concept of self. Conflicts in our mental energies manifest themselves as conflicts with society and the natural environment. For example, when we buy meat products, the id will smell the aroma and feel hungry. The ego will think about price, health and nutrition, while the superego will consider issues related to morality, such as the ethical issue of the deprivation of animal life and the environmental issue of increasing carbon emissions during the process of processing. In the process of this three-self struggle at the individual level, the self at the social level will also play a role: For example, buying or not buying will result in the enrichment or deprivation of the material self and bring pleasure or guilt to the spiritual self, etc. As one level of belief increases, another level of belief weakens. When the superego wins, the ego will reasonably view the gains and losses to the self at the social level and then move forward toward the ecological self and achieve sustainable development.

**Figure 1:**
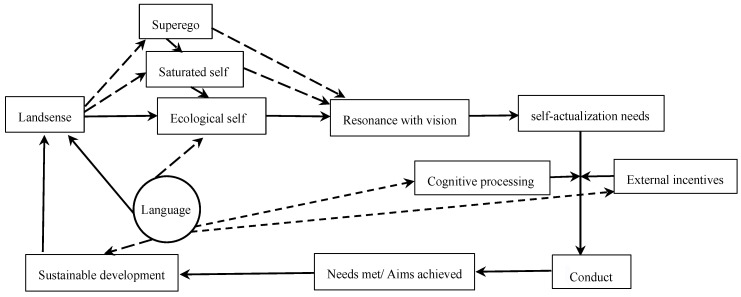
The psychological perception process of landsense creation.

Information about the self is accompanied by information about the environment, and the two are inseparable. Perception has two poles, subjective and objective [13]. By connecting objective physical senses and subjective psychological perceptions, landsenses ecology enables a person to perceive himself/herself as well as the environment. In order to achieve the goal of sustainable landsense creation, “we need to adopt direct expression, indirect expression, metaphor and other associated approaches for landsense creation according to the principles of landsense creation and the actual conditions and practicalities” [11]. Language can effectively describe the self and promote self-cognition and self-realization in the process of landsense creation. As shown in Figure 1 below, the landsense carrying the vision of sustainable development not only provides moral values for the spiritual superego but also provides norms for various social selves (i.e., the saturated self) and then indirectly or directly calls upon the ecological self, promoting sustainable vision resonance and the self-actualization needs that are the highest level of Maslow’s hierarchy of needs. Under the action of cognitive processing and external inducement, self-actualization needs form an internal driving force and contribute to sustainable behavior. Self-actualization needs are met while promoting sustainable development, which is itself the vision embodied in the landsense. Therefore, the psychological perception mechanism of landsense creation constitutes a “meliorization model” [1]. During the process of meliorization, language can not only be a kind of landsense itself, such as beneficial discourse, but also help describe other types of landsense to shape the ecological self and tell the story of sustainable development or to promote the transition from self-actualization needs to ecological behavior by guiding cognitive processing and constructing positive external inducements. Cognitive processing includes self-efficacy, sympathy, empathy and other factors and external incentives include measures such as rewards and punishments.

Social constructionism views self in a new manner by replacing the essential self with the relational self and taking the self to be the product of language [14,15]. Although we tend to think that we control language, it would be more accurate to say that language controls us [16]. From this perspective, the self is not treated as an empirical psychological entity, but as a construction jointly created by culture, society and individuals at a specific time and place. In this process, harmonious discourse analysis (henceforth HDA) should play a positive role in promoting landsense creation.

### 2.3. The Landsense Creation of Harmonious Discourse Analysis and Perceived Self-Efficacy

HDA is a new ecolinguistic research method proposed for the Chinese context by Chinese scholar Guowen Huang (2016/2017) [3,4].

HDA aims to work on two levels: the micro level, which is a text-based level analyzing features and patterns in language forms; and the macro level, which is a translinguistic level analyzing the language system and other systems, whether semiotic, social or material, in terms of their interactions in social praxis. HDA does not merely confirm or criticize a phenomenon, ecosophy or action, but also show how various relations in the ecosystem are harmonized and how language and other systems contribute to harmonizing such relations [17].

HDA promotes ecological education, helps shape social practices and develops new social harmony, which is a process of landsense creation. The theories and methods of landsenses ecology help to reveal “how our ways of meaning affect the impact we have on the environment” [18]. On the one hand, vision-resonance explains how language creates a harmonious vision and arouses social resonance so as to reach an artistic consensus of “do as you would be done by” [1]. On the other hand, resonance-conduct reveals how language inspires people’s environmental self-efficacy and promotes the unity of their awareness and behavior. Environmental self-efficacy belongs to the concept of the Perceived Self-Efficacy (henceforth PSE). In psychology, PSE refers to an individual’s beliefs as to whether he or she will have the ability to organize and manage certain behaviors under certain circumstances in the future. Environmental self-efficacy is an extension of this theory to the field of ecology and refers to an individual’s cognition of his or her ability to reduce the threat of the ecological consequences of his or her behavior. Landsense creation in HDA guides people to recognize the consequences of their own behavior, thus forming an internal control and drive to reduce the threat of their own behavior and then promoting “thinking and acting ecolinguistically” [3].

Kinnear et al. (1974) found consumers would show more concern for ecology as they perceived individuals to be increasingly effective in pollution abatement. Webster (1975) [19] further pointed out that the socially conscious consumer would feel strongly about the fact that he or she could do something about pollution and would try to consider the social impact of his or her purchases. Henion (1976) [20] and Tucker (1978) [21] also found that consumers with an internal locus of control were more inclined to show socially responsible attitudes and behaviors. Roberts (1996) [22] showed that perceived consumer effectiveness (PCE) and environmental concern (henceforth EC) had a positive impact on ecologically conscious consumer behavior (ECCB). The Value-Belief-Norms Theory contained in these arguments, as well as in the Vision-Resonance-Common code of conduct in landsense creation, reveals the causal chain from the ecological self to environmental behavior. The integration of the two can not only reveal the macroscopic process in which language constructs society but also help to quantify environmental self-efficacy and the spillover effect contained in harmonious discourse, as well as further elaborate the psychological perception mechanism of landsenses ecology.

Based on the above review of the relationship between the concept of self, perceived self-efficacy and environmental behavior, as well as the analysis of landsense creation through harmonious discourse, this paper compares the construction of awareness and behavior of low-carbon lifestyles in three environmental publicity texts. An exploratory factor analysis is also conducted into the creation of ecological discourse on environmental behavior decisions (henceforth EBD) in order to reveal the psychological perception mechanism of vision resonance and to provide reference for the construction of ecological discourse on ecological behavior.

## 3. Methods and Procedures

### 3.1. Questionnaire Design and Research Methods

We collected and screened material about low-carbon publicity available on the internet, modified and edited them according to our research purpose and then organized three texts on low-carbon lifestyle.

Each text has roughly the same number of words (296, 270 and 419 characters in Chinese, respectively) and the same structure. It is composed of a brief introduction to a low-carbon lifestyle and 12 low-carbon slogans, but the emphasis of each text is different (See the Appendix A). The introduction to a low-carbon lifestyle in the first text focuses on describing it as a fashionable lifestyle and the following slogans are mainly catchwords for pursuing a low-carbon lifestyle, such as “Practice a low-carbon lifestyle to embrace green fashion” and “Strive to be a guardian of energy conservation and emission reduction; be happy to be a master of low-carbon environmental protection”. The introduction to a low-carbon lifestyle in the second text focuses on the social responsibility of a low-carbon lifestyle, followed by a specific low-carbon code of conduct, such as “Use the elevator as little as possible”, “Promote e-government as much as possible” and “Reduce unnecessary mileage”. The third text focuses on the harm of carbon emissions and attaches a reduction in carbon emissions to each low-carbon code of conduct, such as “Not taking the elevator once reduces carbon emissions by 0.218 kg”, “Skipping fast food once reduces carbon emissions by 0.48 kg” and “Saving 1 km driving reduces carbon emissions by 0.22 kg”.

The study’s questionnaire consists of three sections. The first section has only one item, i.e., “can you practice a low-carbon life after reading this text about low-carbon lifestyle?”, which is used to investigate the effect of discourse on EBD. The choices are organized on a 5-point Likert scale, ranging from 1 point (not at all) to 5 points (absolutely able). The score of this question is used to detect the effect of low-carbon harmonious discourse on EBD.

The second section consists of 12 items (see Table 1), which are used to investigate the subjects’ reasons for making EBD and their psychological perception of the text. The choices are all on a 5-point Likert scale. The scores in this part are used to conduct an exploratory factor analysis on the psychological perception of landsense creation of harmonious discourse.

The third section is used to investigate the subjects’ basic demographic information, including age and sex. The purpose of this study is to verify the degree of vision resonance of low-carbon texts and to explore its influencing factors.

### 3.2. Data Acquisition and Analysis Methods

In this study, 180 college students in the same year of study were randomly selected from an undergraduate university and divided into three groups. Each group read a text about a low-carbon lifestyle and answered questions. A total of 179 valid questionnaires were collected with a response rate of 99.44% (M_age_ = 20.12, SD = 0.95). This study adopted the SPSS 22.0 (IBM Corp., Armonk, NY, USA) for data analysis.

## 4. Results and Discussion

### 4.1. Descriptive Statistics and One-Way Analysis of Variance

The EBD of the subjects is an important manifestation of the creation effect of low-carbon discourse as a landsense. The positive effect of landsense creation is manifested in high EBD, while the negative effect of landsense creation is manifested in low EBD. Therefore, descriptive statistics were conducted on the EBD of three groups of subjects. In terms of whether they could practice a low-carbon lifestyle, 83.33% of the subjects in the first group had a “more likely” attitude and 8.33% had an “absolutely able” attitude, accounting for 91.67% in total. In the second group, the proportions of “more likely” and “absolutely able” were 33.33% and 63.33%, respectively, accounting for 96.67% in total. In the third group, the proportions of “more likely” and “absolutely able” were 61.02% and 10.17%, respectively, accounting for 71.19% in total. The results showed that the three groups of subjects all achieved a better effect of landsense creation. That is, all three texts effectively manipulated the subjects to produce positive EBD.

The EBD of the subjects was selected as the dependent variable and the three texts were selected as the independent variable. A one-way ANOVA test was conducted. The significance level of Levene was 0.137 (greater than 0.05), indicating that the subjects in the three groups were normally distributed and had equal variances, i.e., homogeneity of variance, which provided the basic conditions for inter-group comparative analysis. In other words, the homogeneity test results (Table 2) met the prerequisites of a one-way ANOVA test and could be further used to test whether the mean values of the three groups were equal or different.

A robustness test of mean equivalence (Table 3) showed that the original hypothesis of mean equivalence of the three groups was not valid. The analysis of variance (Table 4) further showed that there were significant differences among the three groups. The F-statistic from the ANOVA test was 29.033, which was much higher than the threshold (i.e., F_0.05_(2,176) = 3.047), and the significance level *p* < 0.001. The results of the one-way ANOVA test showed differences between the three groups in the degree of creation of EBD.

Multiple comparisons (Table 5) showed that there were significant differences in EBD between Groups 1 and 2 and between Groups 2 and 3, but there was no significant difference between Groups 1 and 3. Among them, Group 2 had the highest level of EBD, which was significantly different from the other two groups. Group 1 ranked second in EBD, while Group 3 had the lowest level of EBD.

### 4.2. Exploratory Factor Analysis

Exploratory factor analysis (henceforth EFA) was proposed by Charles Spearman in 1904. Its basic principle is that by means of dimensionality reduction in multivariate statistical analysis, multiple sets of observed variables with high correlation and semantic overlap can be synthesized into a few potential independent factors [23]. Using EFA, not only can the internal relationship between the observed variables and potential independent factors be shown but also a few independent factors thus obtained can be used to replace all the observed variables in order to analyze the EBD of the subjects and the statistical bias caused by the covariant relationship between the factors can be eliminated.

**Figure 2:**
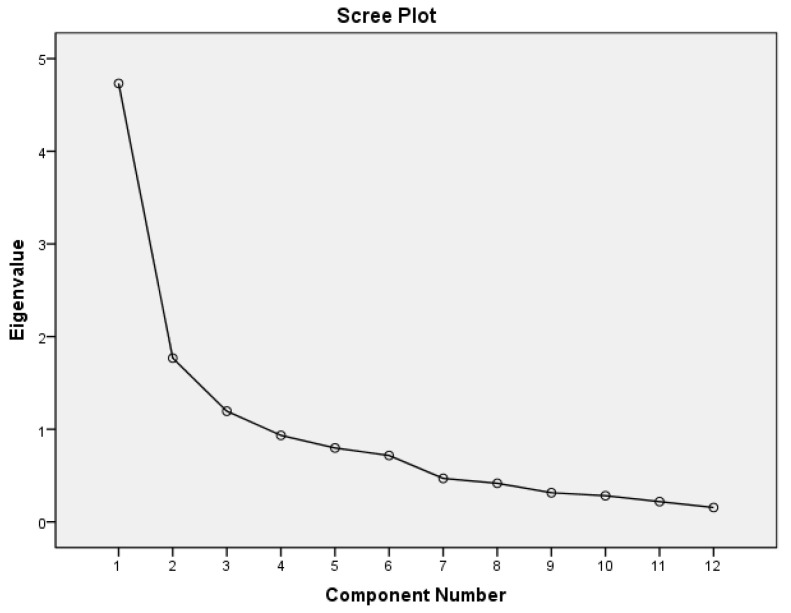
Factor analysis results: Scree plot.

The statistical results of EFA showed that the KMO of the Bartlett sphericity test was 0.753 and the inflection point with eigenvalue greater than one was located in the third dimension (Figure 2). This indicated that the internal dimension of observed variables influencing EBD included three common factors (denoted as F1, F2 and F3, respectively). Variable X1 could be explained by both F1 and F2, but F2 had more explanatory power than F1 (0.602 Vs. 0.522) and so it was put into F2. Thus, the compositions of the three factors were respectively expressed as follows: F1 = f(*X*_2_, *X*_7_, *X*_8_, *X*_9_, *X*_10_, *X*_11_, *X*_12_), F2 = f(*X*_1_, *X*_3_, *X*_4_) and F3 = f(*X*_5_, *X*_6_). Their interpretation rates of variance were 30.441%, 19.024% and 14.659%, respectively, and the cumulative interpretation rate of variance was 64.123% (Table 6 and Table 7). EFA was conducted to find the internal components affecting the EBD of the subjects. When the key words of the original variables were extracted it was discovered that the variables (i.e., *X*_2_, *X*_7_, *X*_8_, *X*_9_, *X*_10_, *X*_11_ and *X*_12_) contained in Factor 1 were mainly related to subjects’ psychological perceptions involving Perceived Self-Efficacy (PSE) about their relationship to a low-carbon lifestyle. The variables contained in Factor 2 (i.e., *X*_1_, *X*_3_ and *X*_4_) mainly measured the subjects’ perception of a low-carbon lifestyle, which belonged to environmental knowledge (EK), while the variables *X*_5_ and *X*_6_ contained in Factor 3 were about the necessity of living a low-carbon lifestyle, suggesting that the EBD was driven by environmental concerns (EC). The meaning of the observed variables contained in the three common factors overlapped and the factors were renamed: F1 = PSE, F2 = EK and F3 = EC. The results of EFA showed that the subjects’ EBD was influenced by PSE, EK and EC induced by the low-carbon harmonious discourse. The proportion of the variance contribution rate of each factor in the total variance contribution rate of the three factors was weighted and summarized and the comprehensive score F of each subject was obtained (see Table 6 and Table 7).

The Cronbach coefficient and Spearman correlation analysis were used to test the internal reliability and structural validity of the three common factors. The internal consistency reliability coefficients (Cronbach’s α1 = 0.847, α2 = 0.763, α3 = 0.538) of the three-dimensional subscales were all in the high reliability or acceptable interval, which indicated that the EBD of the subjects was influenced by their PSE, EK and EC. The overall reliability coefficient Cronbach’s α was 0.852, indicating that the indexes of the questionnaire measuring EBD basically covered all the information.

The results of structural validity test showed that the absolute value of the correlation coefficient between the three dimensions was 0 (showing no correlation), indicating that the difference between the dimensions was significant and that none of the factors could be replaced. The correlation coefficient between the three common factors and the comprehensive score F was between 0.342 and 0.768 (see Table 8) and showed a medium and high correlation, which indicated that the three dimensions were consistent with the overall concept and further proved that the whole scale had good structural validity.

### 4.3. Landsense Creation of Low-Carbon Harmonious Discourse and Climate Change and Health

Landsense creation follows the vein-compliance principle of orientation and bearing. That is, “the position and direction of landsense elements can be ‘subjectively set’ according to the characteristics of the carrier and related ecosystem, the veins of mountain, river (water) and associated fields, the vein of vision presentation (psychological vein), and especially the needs for spatial distribution of landsense elements” [11]. The three texts on low-carbon lifestyles have different psychological veins of landsense elements, but they all carry the vision of a low-carbon lifestyle to protect the environment. A one-way ANOVA test showed that although there were differences in EBD among the three groups, all three texts effectively manipulated the subjects to make positive EBD, which basically reached the resonance of their vision of a low-carbon lifestyle. The 12 items in Table 1 investigated the psychological perception mechanism of each subject in relation to this low-carbon vision. The EFA results showed that the first factor affecting the psychological perception of subjects could be classified as PSE (i.e., F1), which proved that PSE stimulated by ecological discourse helped people form their EBD. The second factor was EK (i.e., F2) contained in the ecological discourse, which meant that improving the affordance of ecological knowledge in discourse was an effective means to help people to reach the resonance of a low-carbon vision and environmental behavior decisions. The third factor affecting the psychological perception of a low-carbon lifestyle could be attributed to EC (i.e., F3), which proved that EC induced by ecological discourse could promote normative behavioral intentions. These findings can effectively guide the landsense creation of harmonious discourse, promote people to practice low-carbon lifestyles and reduce carbon emissions, thus improving the effectiveness of climate change publicity and education.

Greenhouse gas emissions from human activities have reached an all-time high (IPCC 2014) [24]. Urgent action to combat climate change and its impacts is included in the 17 United Nations Sustainable Development Goals (SDGs). The concentration of carbon dioxide in the atmosphere is closely linked to changes in the Earth’s temperature, so reducing carbon dioxide emissions is key to achieving climate action goals. From the point of view of national strategy, improving energy efficiency and saving energy is the preferred strategy for reducing carbon dioxide emissions, but the impact of low-carbon behavior in people’s daily lives on climate change cannot be ignored. In the process of pursuing their material lives, people may damage the health of the environment and the possibility of sustainable development by emitting carbon dioxide through energy consumption in their production and life. Therefore, “sustainable development needs not only the ‘hard’ support of science and technology but also the corresponding ‘soft’ support of culture and ethics even more” [11]. The process of psychological perception of landsense creation in harmonious discourse is a process of guiding people to extend their self-conceptions, expand their self-perceptions and rebuild their self-boundaries. By guiding people to discover and understand themselves, they can achieve a transformation from material self to ecological self so as to achieve harmonious vision resonance and regulate people’s words and deeds. This study shows that the landsense creation of low-carbon harmonious discourse can effectively promote the resonance of low-carbon vision and low-carbon behavior norms in order to achieve the goal of sustainable development. A large number of studies in the field of environmental behavior show that the factors affecting environmental behavior mainly include knowledge and education, personality, self-efficacy, values, attitudes, concern, place attachment, emotions, social norms and so on [25]. This paper verifies the role of PSE, EK and EC in promoting the resonance of a low-carbon vision. Other influencing factors of environmental behavior require further study. Therefore, the role of language in sustainable development and complex environmental issues can be extensively studied from the perspective of the sociality of language.

## 5. Conclusions

Based on the basic principles of landsenses ecology, the concept of linguistic landsense is proposed in this study. A linguistic landsense refers to a meaning carrier that contains one or more of speakers’/designers’ visions through appropriate forms of manifestation, through which listeners/readers can graft, understand and resonate with the visions, thus forming a common code of conduct. It is a theoretical extension of landsenses ecology in the field of semiotic studies and a methodology of ecolinguistics. The results of this study show that discourse containing a low-carbon vision forms a linguistic landsense. The PSE, EK and EC created by the discourse have good explanatory power for the subjects’ environmental behavior decisions, revealing the psychological perception mechanism of the subjects’ achieving the resonance of a low-carbon vision. The specific conclusions are as follows.

(1) The three texts of low-carbon lifestyles all convey the vision of low-carbon harmony and can also form the vision resonance of low-carbon lifestyles in the subjects. The exploratory factor analysis shows that the psychological perception mechanism of landsense creation in low-carbon harmonious discourse can be attributed to three factors: perceived self-efficacy (PSE), environmental knowledge (EK) and environmental concern (EC). PSE has the greatest influence on EBD, followed by EK and EC. PSE reflects individuals’ perception of the ability to reduce the threat of their own behavior consequences. EC reflects individuals’ perception of the necessity for adopting low-carbon behaviors and EK reflects individuals’ perception of specific low-carbon lifestyle events. PSE, EK and EC are the important factors in cognitive processing and are the three pillars underpinning the transition from low-carbon vision to a low-carbon code of conduct. These three factors well describe people’s overall perception and evaluation of low-carbon harmonious discourse. EC triggered by the discourse can promote the resonance of the subjects’ low-carbon vision. EK contained in the discourse is the basis for the transformation of low-carbon vision into low-carbon behavior and PSE is the driving force for achieving the resonance of vision with action, which is conducive to the habituation and automation of low-carbon behavior.

(2) PSE, EK and EC stimulated by low-carbon discourse can promote the subjects’ EBD, which is consistent with the research of Maloney, Ward and Lepisto et al. Lepisto (1974) found that EC was an important predictor of ecologically conscious consumer behavior (ECCB) and Antil (1984) [26] also believed that if one was concerned about the environment, then this concern could result in more ECCBs. PSE has been regarded by psychologists as the best variable in social cognitive theory to predict individual behavior [27], but previous studies mostly focused on the relationship between people’s inherent static PSE, EK, EC and environmental behavior and did not explore the generating process of these three factors in people’s psychology. As early as 1973, Maloney and Ward found that most people had a relatively high degree of verbal commitment and affect to environmental protection, with lower levels of actual commitment and knowledge [28]. Therefore, they advocated developing more knowledge of ecology, environment and pollution in the population. The discourse of ecological harmony is just such an effective way to improve the ecological cognition of the entire population. The three texts in this paper describe a low-carbon lifestyle as a fashionable lifestyle, a responsible lifestyle, or a desperately needed lifestyle and create a low-carbon vision from different psychological veins. Subsequent slogans, whether they are slogans in the narrow sense, or a mix of guidelines and effects, also inspire people’s PSE and EC from different psychological perspectives as well as conveying knowledge and visions of low-carbon lifestyles. Subjects grasp this low-carbon vision from these texts and gain a sense of self-efficacy and the corresponding environmental knowledge and concern from the expressions that conform to their own psychological veins so as to achieve the vision resonance of low-carbon lifestyles, make corresponding environmental behavior decisions and regulate their own words and deeds.

(3) Due to limited sample collection, the three low-carbon texts were not expected to have exactly the same ANOVA results among different subjects. That is, differences between groups may vary among different subjects because the psychological perceptions and psychological veins of different subjects may vary. This paper only discusses and analyzes three factors that affect the landsense creation of harmonious discourse. However, the use of these factors to enhance harmonious vision resonance in different populations requires further research.

To sum up, on the one hand this study reveals that the landsense creation of harmonious discourse is an effective method for transforming PSE, EK and EC into environmental behavior. Government agencies, communities, collectives or individuals can take these three factors into consideration in environmental awareness-raising campaigns to effectively enhance public awareness of environmental protection and to improve the publicity effect of environmental protection. On the other hand, it proves that the causal chain of ecological self—vision resonance—self-actualization needs—conduct is the psychological perception mechanism for harmonious discourse to reach vision resonance. However, it is important to note that mental cognition is “an endless process of accumulation and deepening. So is the cognition to ‘sense’ and the functional mechanism of sense” [11]. Different people possess different sensitivities to physical elements and the perception of psychological elements and the triggers of PSE are also different. Future research can further investigate the characteristics of subjects’ groups and seek more targeted strategies to stimulate people’s self-efficacy and promote the occurrence of sustainable behaviors.

## Figures and Tables

**Table 1:** Items for factor analysis.

Code	Variables	1	2	3	4	5
*X*_1_	Do you understand the concept of low-carbon lifestyles after reading this text?	not at all	not very	uncertain	very	extremely
*X*_2_	Do you understand the significance of low-carbon lifestyles after reading this text?	not at all	not very	uncertain	very	extremely
*X*_3_	Do you know what should be done for a low-carbon lifestyle after reading this text?	not at all	not very	uncertain	very	extremely
*X*_4_	Do you know what you can do for a low-carbon lifestyle after reading this text?	not at all	not very	uncertain	very	extremely
*X*_5_	Do you think it is important to live a low-carbon life after reading this text?	not at all	not very	uncertain	very	extremely
*X*_6_	Does this text give you a strong sense of environmental urgency?	not at all	not very	uncertain	very	extremely
*X*_7_	Does this text give you a strong sense of social responsibility?	not at all	not very	uncertain	very	extremely
*X*_8_	Do you think low carbon is closely related to daily life after reading this text?	not at all	not very	uncertain	very	extremely
*X*_9_	Do you think personal behavior is closely related to environmental protection after reading this text?	not at all	not very	uncertain	very	extremely
*X*_10_	Do you aspire to a low-carbon lifestyle?	not at all	not very	uncertain	very	extremely
*X*_11_	Will you feel guilty about wasteful behavior later?	not at all	not very	uncertain	very	extremely
*X*_12_	Will you pay attention to the details of low-carbon lifestyles in the future?	not at all	not very	uncertain	very	extremely

**Table 2:** Test of homogeneity of variances.

Levene Statistic	df1	df2	Sig.
2.014	2	176	0.137

**Table 3:** Robustness tests of the equality of means.

	Statistic ^a^	df1	df2	Sig.
Brown–Forsythe	28.213	2	153.064	0.000

a. Asymptotically F distributed.

**Table 4:** ANOVA.

	Sum of Squares	df	Mean Square	F	Sig.
Between Groups	21.772	2	10.886	29.033	0.000
Within Groups	65.993	176	0.375		
Total	87.765	178			

**Table 5:** Multiple Comparisons.

(I) Groups	(J) Groups	Mean Difference (I–J)	Std. Error	Sig.	95% Confidence Interval	
					Lower Bound	Upper Bound
Group 1	Group 2	−0.68000 *	0.11725	0.000	−0.9114	−0.4486
	Group 3	0.09391	0.11373	0.410	−0.1305	0.3184
Group 2	Group 1	0.68000 *	0.11725	0.000	0.4486	0.9114
	Group 3	0.77391 *	0.10809	0.000	0.5606	0.9872
Group 3	Group 1	−0.09391	0.11373	0.410	−0.3184	0.1305
	Group 2	−0.77391 *	0.10809	0.000	−0.9872	−0.5606

*. The mean difference is significant at the 0.05 level.

**Table 6:** Total variance explained.

Component	Initial Eigenvalues			Extraction Sums of Squared Loadings			Rotation Sums of Squared Loadings		
	Total	% of Variance	Cumulative %	Total	% of Variance	Cumulative %	Total	% of Variance	Cumulative %
1	4.732	39.437	39.437	4.732	39.437	39.437	3.653	30.441	30.441
2	1.768	14.730	54.167	1.768	14.730	54.167	2.283	19.024	49.464
3	1.195	9.956	64.123	1.195	9.956	64.123	1.759	14.659	64.123
4	0.934	7.784	71.907						
5	0.798	6.648	78.556						
6	0.716	5.969	84.525						
7	0.469	3.911	88.436						
8	0.417	3.472	91.908						
9	0.314	2.621	94.529						
10	0.283	2.357	96.886						
11	0.218	1.819	98.705						
12	0.155	1.295	100.000						

Extraction method: Principal component analysis.

**Table 7:** Rotated Component Matrix (showing only loads greater than 0.5).

	Component		
	1	2	3
*X*_9_ (individual and environmental protection)	0.832		
*X*_10_ (aspirations)	0.754		
*X*_7_ (responsibility)	0.717		
*X*_8_ (low carbon and daily life)	0.667		
*X*_12_ (details of life)	0.632		
*X*_2_ (significance)	0.580		
*X*_11_ (waste)	0.527		
*X*_4_ (what can be done)		0.887	
*X*_3_ (what should be done)		0.850	
*X*_1_ (concept)	0.522	0.602	
*X*_5_ (importance)			0.896
*X*_6_ (urgency)			0.569

Extraction Method: Principal Component Analysis.; Rotation Method: Varimax with Kaiser Normalization; a. Rotation converged in 5 iterations.

**Table 8:** Correlations.

			F1	F2	F3	F
Spearman’s rho	F1	Correlation Coefficient	1.000			
		Sig. (2-tailed)	0.000			
	F2	Correlation Coefficient	−0.015	1.000		
		Sig. (2-tailed)	0.840	0.000		
	F3	Correlation Coefficient	−0.009	−0.007	1.000	
		Sig. (2-tailed)	0.900	0.922	0.000	
	F	Correlation Coefficient	0.768 **	0.342 **	0.400 **	1.000
		Sig. (2-tailed)	0.000	0.000	0.000	0.000

**. Correlation is significant at the 0.01 (2-tailed).

## Data Availability

The data presented in this study are available on request from the corresponding author. The data are not publicly available due to insert reason here.

## Appendix A. The Following are the Three Groups of Publicity Texts about Low-Carbon Lifestyles Used in this Experiment

Group 1:

A low-carbon lifestyle represents a healthier, more natural, safer and more environmentally friendly lifestyle. At the same time, it is also a low-cost way of life and a lifestyle in line with the trend of the times. For this purpose, we need to conduct ourselves in the following manner.

1. Practice a low-carbon lifestyle to embrace green fashion;
2. Strive to be a guardian of energy conservation and emission reduction; be happy to be a master of low-carbon environmental protection;
3. Cherish every piece of paper and save every kilowatt of electricity;
4. Practice energy-saving behavior bit by bit to achieve a low-carbon home;
5. Start from me, energy saving, low carbon and healthy;
6. Reject the greenhouse effect and embrace a low-carbon life;
7. Promote the green office and practice resource conservation;
8. Promote energy conservation and low carbon to achieve circular development;
9. Use energy-saving products and low-carbon life starts from me;
10. Low carbon leads the environmental fashion and a quality life starts from me;
11. Participate in energy conservation and emission reduction to build a better future;
12. Choose a low-carbon lifestyle and a healthy lifestyle.

Group 2:

A low carbon lifestyle represents a kind of attitude for the sake of a greater number of people and the earth belongs to all life on earth rather than just to individuals. A low-carbon lifestyle is the attitude that we are responsible for in order to achieve a better life in the future. It is also a green, healthy and environmentally friendly way of life.

1. Use the elevator as little as possible;
2. Promote e-government as much as possible;
3. Reduce smoking;
4. Save water;
5. Reduce unnecessary mileage;
6. Use energy-saving lights;
7. Reduce the use of disposable goods;
8. Reduce the use of products containing benzene solvents;
9. Turn off the power when the air conditioner is not in use;
10. Use recycled paper whenever possible;
11. Eat less meat and more vegetables;
12. Stop wasting food.

Group 3:

The increase in carbon emissions is a direct cause of natural disasters, such as the greenhouse effect of global warming and extreme weather. Scientists predict that if the Earth’s surface temperature continues to rise at the current rate, by the year 2050, icebergs in the north and south polar regions will have melted and sea levels will rise so much that some island countries and coastal cities will disappear. Therefore, we should practice a low-carbon lifestyle and prevent global warming.

1. Not taking the elevator once reduces carbon emissions by 0.218 kg;
2. Reducing the use of air conditioning by 1 h can reduce carbon emissions by 0.621 kg;
3. Reducing the use of electric fans for 1 h can reduce carbon emissions by 0.045 kg;
4. One hour less time spent watching TV can reduce carbon emissions by 0.096 kg;
5. Reducing the use of incandescent bulbs for one hour can reduce carbon emissions by 0.041 kg;
6. Saving 1 km driving reduces carbon emissions by 0.22 kg;
7. Skipping fast food once reduces carbon emissions by 0.48 kg;
8. One kilogram less garbage can reduce carbon emissions by 2.06 kg;
9. Saving 1 ton of water can reduce carbon emissions by 0.194 kg;
10. Cutting back on the electric treadmill for 45 min can save nearly 1 kg of carbon dioxide emissions;
11. Less waste of a small bowl of rice can reduce carbon dioxide by 0.09 kg;
12. A vegetarian diet reduces carbon emissions in one day by the equivalent of the carbon dioxide absorbed by 150–350 trees; A vegetarian diet is equal to planting 180 trees a day.
